# Air pollution induces *Staphylococcus aureus* USA300 respiratory tract colonization mediated by specific bacterial genetic responses involving the global virulence gene regulators Agr and Sae

**DOI:** 10.1111/1462-2920.16076

**Published:** 2022-06-01

**Authors:** Jo Purves, Shane J. K. Hussey, Louise Corscadden, Lillie Purser, Andie Hall, Raju Misra, Liza Selley, Paul S. Monks, Julian M. Ketley, Peter W. Andrew, Julie A. Morrissey

**Affiliations:** ^1^ Department of Genetics University of Leicester, University Road Leicester LE1 7RH UK; ^2^ Molecular Biology, Core Research Laboratories Natural History Museum Cromwell Road, London SW7 5BD UK; ^3^ MRC Toxicology Unit University of Cambridge Cambridge CB2 1QR UK; ^4^ Department of Chemistry University of Leicester University Road Leicester LE1 7RH UK; ^5^ Department of Respiratory Sciences University of Leicester University Road, Leicester LE1 9HN UK

## Abstract

Exposure to particulate matter (PM), a major component of air pollution, is associated with exacerbation of chronic respiratory disease, and infectious diseases such as community‐acquired pneumonia. Although PM can cause adverse health effects through direct damage to host cells, our previous study showed that PM can also impact bacterial behaviour by promoting *in vivo* colonization. In this study we describe the genetic mechanisms involved in the bacterial response to exposure to black carbon (BC), a constituent of PM found in most sources of air pollution. We show that *Staphylococcus aureus* strain USA300 LAC grown in BC prior to inoculation showed increased murine respiratory tract colonization and pulmonary invasion *in vivo*, as well as adhesion and invasion of human epithelial cells *in vitro*. Global transcriptional analysis showed that BC has a widespread effect on *S*. *aureus* transcriptional responses, altering the regulation of the major virulence gene regulators Sae and Agr and causing increased expression of genes encoding toxins, proteases and immune evasion factors. Together these data describe a previously unrecognized causative mechanism of air pollution‐associated infection, in that exposure to BC can increase bacterial colonization and virulence factor expression by acting directly on the bacterium rather than via the host.

## Introduction

Air pollution is the world's largest single global environmental health risk with an estimated 90% of people worldwide breathing polluted air, this pollution is responsible for over 7 million deaths per year (World Health Organization − News Release, 2018). It is the result of natural and anthropogenic activity, with increased urbanization resulting in significant increases in types and concentrations of pollutants (Manisalidis *et al*., 2020). Particulate matter (PM) is a major component of air pollution, with particles of <2.5 μm causing the most serious adverse health effects due to deposition in the upper respiratory tract and the ability to enter the lower respiratory tract and bloodstream (Cohen *et al*., 2017; McNeil, 2019).

PM exposure is strongly associated with cancer and cardiovascular diseases, and exacerbation of chronic respiratory disease, such as COPD and asthma (Cohen *et al*., 2017). There is also an association with infectious disease, with community‐acquired pneumonia rates most affected (Neupane *et al*., 2010; Qiu *et al*., 2014), but less well known is the impact on infective endocarditis (Hsieh *et al*., 2019), infection of cystic fibrosis patients (Psoter *et al*., 2015; Psoter *et al*., 2017), otitis media (Park *et al*., 2018), chronic rhinosinusitis (Schwarzbach *et al*., 2020) and adverse effects on chronic skin diseases (Dijkhoff *et al*., 2020). High levels of PM exposure also alter respiratory microbiome diversity (Li *et al*., 2017; Li *et al*., 2019; Mariani *et al*., 2018; Mariani *et al*., 2020; Rylance *et al*., 2016; Wang *et al*., 2019).

In seeking to explain how PM adversely affects chronic and infectious diseases, research has focused on direct damage to host tissue caused by PM exposure, including increased inflammation, and oxidative stress (Lee *et al*., 2021). It is also known that in infection, PM can potentiate disease by repressing the immune system (Castranova *et al*., 2001; Liu *et al*., 2019; Migliaccio *et al*., 2013; Shears *et al*., 2020; Yang *et al*., 2001) and by disruption of epithelial function (Liu *et al*., 2019; Misiukiewicz‐Stepien & Paplinska‐Goryca, 2021). The possibility that PM may directly affect bacteria had not received attention until recently. In Hussey *et al*. (2017) we showed that air pollution does have a direct impact on bacterial behaviour.

Direct exposure of *Staphylococcus aureus* and *Streptococcus pneumoniae* to black carbon (BC), a by‐product of biomass burning and a major constituent of PM (Bell *et al*., 2007), results in major changes in bacterial biofilm formation and antibiotic susceptibility (Hussey *et al*., 2017). Additionally, we found that in mice simultaneously exposed to *S*. *pneumoniae* and BC there was increased bacterial dissemination to the lungs (Hussey *et al*., 2017). The instillation of BC into the mice did not cause detectable tissue damage, indicating that BC acts as a signal that alters bacterial behaviour (Hussey *et al*., 2017). Subsequent studies also showed that direct bacterial exposure to a variety of PM sources also increased biofilm formation (Woo *et al*., 2018; Yadav *et al*., 2020) and increased *S*. *pneumoniae* nasopharyngeal colonization and dissemination to the lungs and middle ear of mice (Yadav *et al*., 2020). None of these studies sought to determine the biological mechanisms involved in the bacterial response to PM exposure, but the BC‐induced changes could be a key contributing factor in how air pollutants cause increased lower respiratory tract infectious disease.

Here we report investigation of not only the impact of BC on nasopharyngeal colonization and invasion by the community‐acquired, methicillin‐resistant *S*. *aureus* (CA‐MRSA) strain USA300 LAC but also the genetic mechanisms involved. We show that, relative to *S*. *aureus* alone, simultaneous inoculation of BC and *S*. *aureus* into the nasopharynx increases *S*. *aureus* numbers in the nasopharynx, lungs and blood of mice, and increases *S*. *aureus* adhesion to human respiratory epithelial cells. *Staphylococcus aureus* grown in BC prior to inoculation also showed increased murine respiratory tract colonization and invasion *in vivo* as well as adhesion and invasion of human epithelial cells *in vitro* supporting the hypothesis that BC acts as a hitherto unconsidered signal that has a direct effect on *S*. *aureus* behaviour. Global transcriptional analysis showed that BC does indeed have a widespread effect on *S*. *aureus* transcriptional responses, altering the regulation of the major virulence gene regulators Sae and Agr and causing increased expression of genes for toxins, proteases and immune evasion factors.

## Experimental procedures

### Bacterial strains and growth conditions

The methicillin‐resistant *S*. *aureus* USA300 LAC was used in this study (Kennedy *et al*., 2008). Transduction with Phage 11 was used to move the *bursa aurealis agrB*::Tn (strain ΦΕ95) transposon insertion mutation from the Nebraska Transposon Mutant Library (Bae *et al*., 2008; Fey *et al*., 2013) and the *saeS*::*Tn917* from strain Newman *sae*::*Tn917* (Goerke *et al*., 2001) into USA300 LAC. A double mutant strain LAC *agr*::tet/∆*saePQRS* was kindly provided by A. Horswill (University of Colorado). Mutant strains were confirmed by PCR using gene‐specific primers (Table S1). Unless otherwise stated, bacteria were grown in Tryptic Soy Broth (TSB; Beckton Dickinson) statically at 37°C in 5% vol./vol. CO_2_.

### Black carbon

BC (Sigma‐Aldrich product number 699632) was dispersed in sterile dH_2_O at 2–10 mg ml^−1^. The particle size of the powder was <500 nm and it contained <500 ppm trace metals. BC is an ideal model particulate for this study as BC does not affect bacterial growth (Hussey *et al*., 2017), unlike purified synthetic nanoparticles such as Carbon Black which are generally toxic to bacteria (Al‐Jumaili *et al*., 2017).

### Murine colonization model

Experiments were carried out in accordance with the UK Home Office Project Licence P7B01C07A. Female 8‐week‐old outbred CD1 mice from Charles River, UK were used. Animals were allowed to acclimatize for 1 week prior to the experiments. Animals were housed in groups of five, maintained on a 12 h dark/light cycle and allowed unrestricted access to food and water. Prior to use, bacteria were grown in TSB in the presence and absence of 100 μg ml^−1^ BC, to mid‐exponential phase, and stored in aliquots at −80°C. For use, frozen aliquots were thawed, washed and resuspended in PBS. Mice were intranasally infected with 15 μl containing 1 × 10^7^ CFU *S*. *aureus* USA300, or 1 × 10^7^ CFU LAC mixed with 105 μg of BC as previously described (Hussey *et al*., 2017). After infection, the mice showed no signs of disease over the following 7 days. At days 1 and 7 post‐infection the numbers of bacteria in the nasopharynx, lungs and blood of preselected animals were assessed in homogenized tissue by serial dilution and plating (Hussey *et al*., 2017). Significance was determined using a Kruskal–Wallis test with Dunn's multiple comparison.

### RNA extraction

*Staphylococcus aureus* strains were grown to late‐exponential phase in TSB, with and without 100 μg ml^−1^ BC. To preserve RNA integrity, cultures were treated with RNAprotect (Qiagen) and cells were pelleted and stored at −80°C as per the manufacturer's instructions. Bacteria were lysed in 200 μl Tris EDTA (TE) buffer containing 100 μg ml^−1^ lysostaphin and 50 μg ml^−1^ proteinase K final concentration. 600 μl of Trizol reagent was added and cells were then mechanically disrupted using an MP Biomedicals FastPrep Instrument and Lysing Matrix B tubes (MP Biomedicals). BC particles were removed by centrifugation at 12 000*g* and RNA extracted from the bacteria in the supernatant using a Direct‐zol RNA Miniprep Plus Kit (Zymogen) following the manufacturer's instructions. Samples were further treated with TURBO DNA‐free (Ambion) to ensure complete removal of DNA, which was confirmed via qPCR. RNA concentrations were determined using a Nanodrop spectrophotometer.

### RNAseq

RNA quality and integrity were assessed using a 2100 Bioanalyser and RNA 6000 Nano chip (Agilent), to ensure a minimum RNA integrity value of 8 (Table S4). Samples were depleted for ribosomal RNA and libraries were prepared using ScriptSeq RNA Library Preparation before paired‐end sequencing on an Illumina NextSeq550.

RNAseq data quality was assessed using FastQC (v. 0.11.5). Trimmomatic (v. 0.36) was used to remove adaptor sequences, and the read correction tool SOAPec (v. 2.01) was used to identify and repair errors in the read data. The reads were mapped to the *S*. *aureus* USA300 FRP3757 genome (accession no. CP000255) using HISAT2 (v 2.1.0) and the transcriptome was assembled using STRINGTIE (v. 1.3.3b). The R package DESeq2 was used to test differential gene expression between the samples, and gene expression is expressed as the Log2 Fold Change (L2FC) in expression relative to growth without BC. The screening threshold for the results was set at >1 or <−1 L2FC using an adjusted *p*‐value (pADJ) of 0.001.

To determine whether any functional groups of genes were significantly upregulated or downregulated in response to BC, GO Enrichment Analysis was carried out and the Fisher's exact test with a *p*‐value ≤0.05 was used to test the enrichment in each category (Ashburner *et al*., 2000; Gene, 2021; Mi *et al*., 2019). Additional gene function data, including TIGRFAM functional groups, were extracted for each locus from the AureoWiki database, which provides a pan‐genome approach to functional annotation of genes (Fuchs *et al*., 2018).

### Quantitative reverse transcriptase PCR

Total RNA was converted into cDNA using Superscript IV VILO Master Mix reverse transcriptase (Invitrogen), and 0.5 ng of cDNA was used for each qPCR reaction. qRT‐PCR was done using SYBR Green Master Mix (Applied Biosystems) in a 7300 Fast System (Applied Biosystems) following the manufacturer's instructions. Relative gene expression for each of the sample genes (for primer details see Table S1) was normalized to the expression of the endogenous control genes *gyrB* and 16S rRNA and expressed relative to the LAC wild‐type strain cultured without BC, using the ∆∆Ct method to calculate RQ (2^−∆∆Ct^) (Livak & Schmittgen, 2001). Significance was determined by a Kruskal–Wallis test with Dunn's multiple comparison test (*p < 0.05, **p < 0.01).

### Alpha‐haemolysin activity assay

To prepare erythrocytes, heparinised rabbit blood was diluted with 20 vol. of PBS and centrifuged at 3000*g* for 5 min to pellet the cells. Erythrocytes were washed once in 20 vol. PBS and then resuspended in 6 vol. PBS. To prepare the bacteria *S*. *aureus* USA300 LAC was grown to late‐exponential phase in TSB, with and without 100 μg ml^−1^ BC. Cultures were centrifuged at 3000*g* for 5 min to pellet the cells, and supernatants were filter sterilized using a 0.2 μm filter membrane. Cell pellets were washed twice in an equal volume of PBS. To measure haemolytic activity an equal volume of prepared erythrocytes and either supernatant or cell suspension were mixed and incubated at 37°C for 30 min. Cells were also mixed with 100 μg ml^−1^ BC suspended in PBS to determine the haemolytic activity of BC alone. Cell suspensions were centrifuged at 120*g* for 7 min to pellet intact erythrocytes. The absorbance of the suspension supernatant was measured at 450 nm. A PBS control was used to measure spontaneous haemolysis and SDS was used to measure total erythrocyte lysis. Percentage of total haemolytic activity was calculated as follows:

$$\begin{array}{l} {}[\left(A450_{\text{sample}}-A450_{\text{spontaneous}}\right)/(A450_{\text{total lysis}}\\ \;-\;A450_{\text{spontaneous}})]\times 100 \end{array}$$

Data are presented as the mean of three independent biological replicates (±SEM) and significance was determined by two‐way ANOVA.

### Epithelial cell adhesion, invasion and persistence

For bacterial adhesion and invasion studies, the human Type II‐like bronchial epithelial cell line A549 was used. 24‐well tissue culture plates were seeded with 1 × 10^5^ cells in RPMI with 1% vol./vol. foetal bovine calf serum (FBS) and grown to 70%–100% confluency prior to inoculation with *S*. *aureus*. A549 cells were inoculated with 1 × 10^7^ CFU of *S*. *aureus* under the following conditions: (i) *S*. *aureus* LAC alone, (ii) *S*. *aureus* LAC plus 100 μg ml^−1^ of BC or (iii) *S*. *aureus* LAC grown in the presence of 100 μg ml^−1^ BC. Bacterial cells were washed twice in PBS and diluted to 1 × 10^7^ CFU/50 μl in PBS prior to A549 inoculation. All doses were confirmed by serial dilution and plating. Infected cells were incubated at 37°C in 5% vol./vol. CO_2_ for 2 h. For adhesion assays, cells were washed in PBS and lysed in 1% vol./vol. Triton‐X‐100 for 10 min. Bacterial CFU was determined by serial dilution and plating. For invasion and persistence assays, 2 h post‐inoculation A549 cells were washed and resuspended in RPMI containing 300 μg ml^−1^ gentamicin for 2 h (invasion) before washing and lysing the cells as described above, followed by serial dilution and plating to determine CFU (Richards *et al*., 2015). Data are presented as the mean of at least three independent biological replicates (±SEM) and significance was determined by one‐way ANOVA with Tukey's multiple comparison test.

### Cytotoxicity

A549 cytotoxicity was measured by the presence of lactate dehydrogenase (LDH) in the culture medium. LDH release was measured using CyQuant LDH Cytotoxicity Assay Kit (Invitrogen) as per the manufacturer's instructions. LDH activity was assayed in supernatant from uninfected cells (spontaneous damage), cells infected with bacteria alone and with 100 μg ml^−1^ BC and cells exposed to 100 μg ml^−1^ BC alone (to determine cell damage from BC specifically). Absorbance was measured at 490 and 680 nm (background) and the background value was subtracted from the 490 nm reading to give LDH activity. % cytotoxicity was calculated as follows:

$$[\left(\text{Supernatant}\;\mathrm{LDH}\;\text{activity}-\text{Spontaneous}\;\mathrm{LDH}\;\text{activity}\right)/(\mathrm{MAX}\;\mathrm{LDH}\;\text{activity}\;-\;\text{Spontaneous}\;\mathrm{LDH}\;\text{activity})]\times 100$$

Data are presented as the mean of three independent biological replicates (±SEM) and significance was determined by one‐way ANOVA with Tukey's multiple comparison test.

## Results

### Exposure of *S*. *aureus* to BC prior to inoculation increases bacterial numbers in the respiratory tract

Previous studies have shown that bacterial dissemination from the nasopharynx to murine or rat lungs is induced when the animals are exposed to different forms of PM before bacterial inoculation or when bacteria and PM are simultaneously inoculated (Hussey *et al*., 2017; Shears *et al*., 2020; Yadav *et al*., 2020; Zhao *et al*., 2014). However, the direct effect of PM on bacterial behaviour during colonization was not fully established because the presence of significant levels of PM within the host could potentiate colonization by several mechanisms including damaging host tissue, by acting as a vehicle to support bacterial dissemination through the respiratory tract, or by supporting bacterial growth.

Our previous work showed that BC increased colonization and invasion by *S*. *pneumoniae*; however, it was not established whether this was due to direct effects on the bacterium and/or to effects on the host. Biologically relevant concentrations of BC are used that are at the lower end of the range of total amounts (96–378 μg day^−1^) of air pollution PM reported to be inhaled and deposited in the human respiratory tract each day (Chalvatzaki *et al*., 2018). To establish if BC directly affects the *in vivo* behaviour of *S*. *aureus*, mice were intranasally inoculated with the bacterium grown in the presence of BC but with the BC particles removed by dilution prior to inoculation. Mice were also inoculated with *S*. *aureus* LAC alone and LAC simultaneously inoculated with BC.

**Fig. 1:**
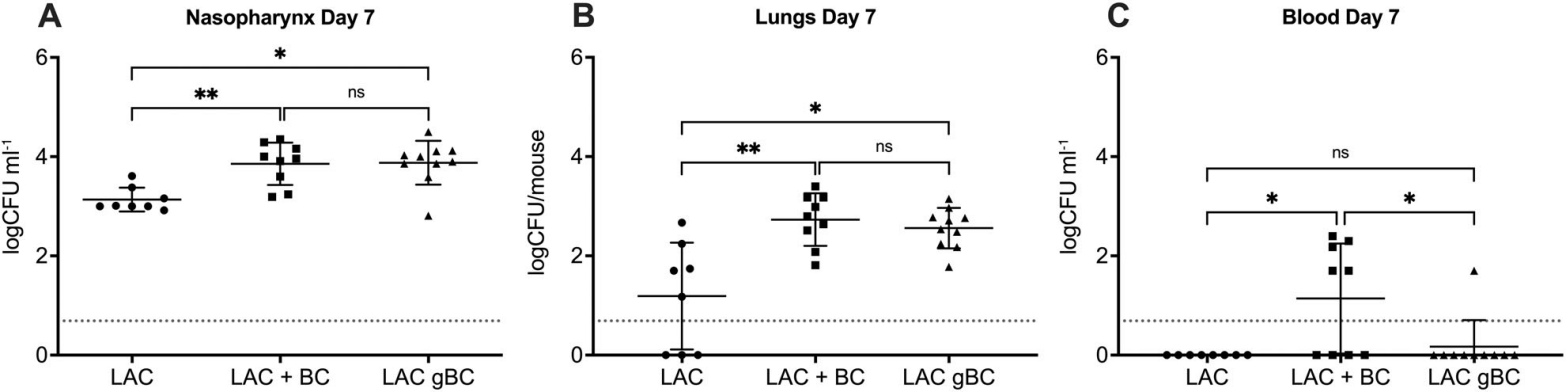
Exposure of *S*. *aureus* to BC results in increased respiratory tract colonization in mice. Female CD1 mice were intranasally inoculated with 15 μl containing 10^7^ *S*. *aureus* LAC (−BC), 10^7^ LAC with 105 μg BC (+BC) or 10^7^ CFU LAC pre‐grown in the presence of 100 μg ml^−1^ BC (gBC). After 7 days, bacteria were recovered from the nasopharynx (A), lungs (B) and blood (C) and plated to determine the bacterial CFUs. No mice showed any clinical signs of infection. Data are presented as logCFU ml^−1^ for nasopharyngeal washes and blood, and logCFU/mouse for lungs. The dotted line marks the limit of detection. Data were analysed using a Kruskal–Wallis test with Dunn's multiple comparison (*p < 0.05, **p < 0.01).

*Staphylococcus aureus* pre‐grown in BC prior to inoculation (gBC) significantly increased staphylococci in the nasopharynx (Figure 1A) and lungs (Figure 1B) at day 7 post‐infection period compared to the control without BC (*p* < 0.05). When *S*. *aureus* were co‐inoculated together with BC (LAC + BC) a significant increase in *S*. *aureus* in the nasopharynx and lungs by 7 days post‐infection was also observed (Figure 1A and B both *p* < 0.01 compared to the control). In contrast, only after co‐inoculation of BC and *S*. *aureus* were there more staphylococci in the blood compared to the BC control (Figure 1C, *p* < 0.05). None of the mice had visible signs of disease and all survived throughout the experiment. Together these data indicate that the increase in infection of the nasopharynx and lungs by *S*. *aureus* is caused by the BC acting directly on the bacterium rather than via the host, but the presence of BC is important for bacterial invasion to the blood.

### *Staphylococcus aureus* pre‐grown in BC show increased adhesion and invasion of epithelial cells *in vitro*

Because *S*. *aureus* can invade non‐professional phagocytes thereby avoiding aspects of the immune system (Garzoni & Kelley, 2008), we determined whether BC alters adherence or invasion of *S*. *aureus* to human respiratory epithelial cells. The type II‐like bronchial epithelial cells, A549, were exposed to *S*. *aureus* LAC grown in the same conditions as the murine colonization.

**Fig. 2:**
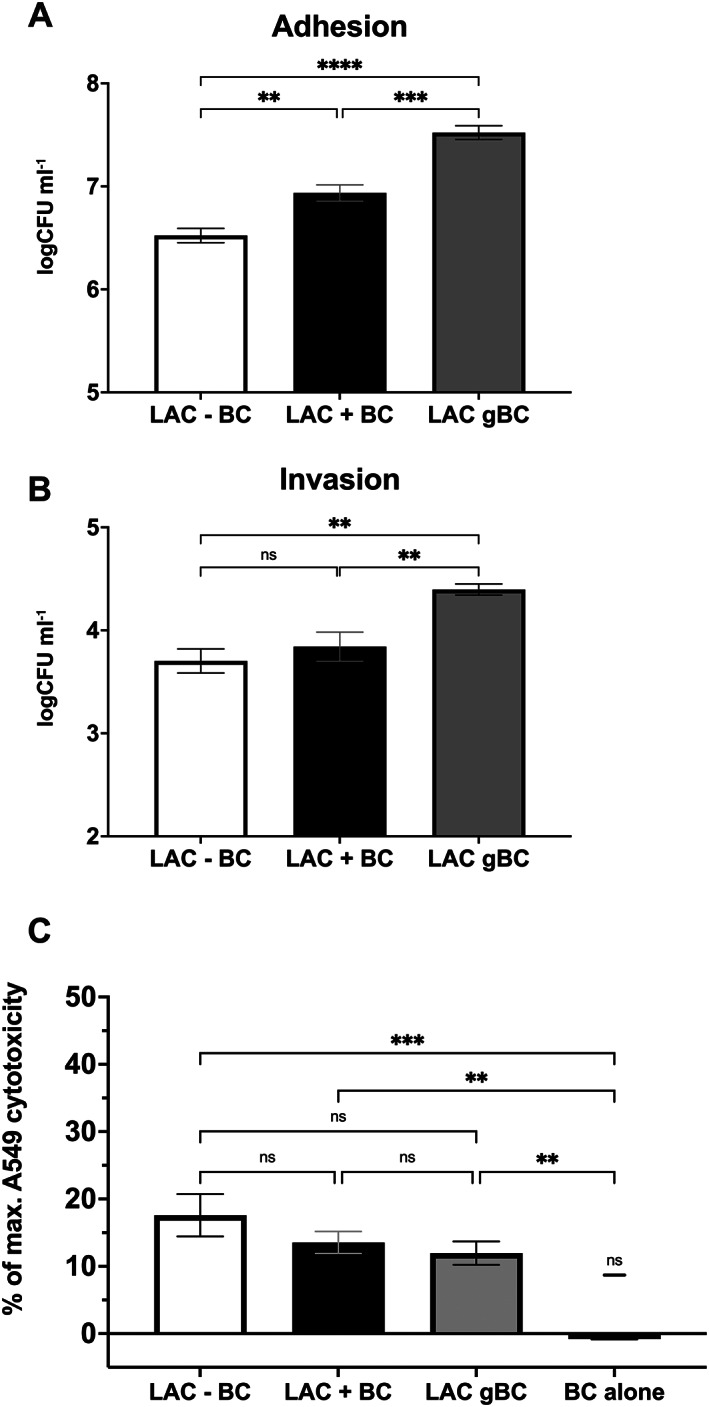
Exposure to BC results in increased adhesion, invasion and persistence within human epithelial cells. *Staphylococcus aureus* LAC adhesion (A), and invasion (B) of human lung epithelial A549 cells was measured using a gentamicin protection assay. Monolayers of 1 × 10^5^ A549 cells in 24‐well plates were infected at a MOI of 100. Data are presented as logCFU ml^−1^ and error bars represent 1 SEM of at least five biological repeats. Significance was determined by one‐way ANOVA with Dunnett's multiple comparison test (*p < 0.05, **p < 0.01, ***p < 0.001, ****p < 0.0001). (C) Cytotoxicity was measured through LDH release from A549 cells after 2 h exposure to *S*. *aureus* and/or BC. % cytotoxicity is calculated relative to spontaneous cell death (0%) and maximum cell death (100% cell lysis). Significance was determined by one‐way ANOVA with Tukey's multiple comparison test (*p < 0.05, **p < 0.01, ***p < 0.001, ****p < 0.0001).

There was a significant increase in numbers of *S*. *aureus* adhering to (Figure 2A, *p* < 0.0001) and invading (Figure 2B, *p* < 0.01) A549 cells when grown with BC (gBC) prior to inoculation compared to bacteria grown in the absence of BC (−BC), an observation consistent with a direct effect of BC on the bacteria. There was also increased adhesion of *S*. *aureus* to A549 cells when simultaneously inoculated with BC (+BC) (Figure 2A, *p* < 0.01) but no significant change in invasion. Importantly inoculation with BC alone did not affect A549 cell viability (Figure 2C). Incubation of A549 with *S*. *aureus* LAC caused 18% cytotoxicity but there was no significant increase in cytotoxicity on A549 cells during co‐inoculation with BC or when *S*. *aureus* were pre‐grown in BC (Figure 2C). Together these data demonstrate that increased *S*. *aureus* epithelial adhesion and invasion is caused by direct interaction of BC with the bacteria and does not involve gross changes to the epithelial cells.

### BC induces expression of *S*. *aureus* genes for toxins and proteases and the SOS response

Having shown that BC alters the phenotype of *S*. *aureus*, we used transcriptome sequencing (RNAseq) to investigate the pattern of gene expression induced using the same BC exposure growth conditions as the colonization experiments. RNAseq analysis identified 52 staphylococcal genes that showed a significant increase in expression (Table 1 and Table S2) and 63 genes that showed a significant decrease in expression (Table 2 and Table S3) after exposure to BC.

**Table 1:** Genes significantly upregulated at a fold change >2 in response to BC.

Locus tag (SAUSA300)	Gene name	Product description	Fold change
RS11190	*kdpA*	Potassium‐transporting ATPase subunit A	6.30
RS09620	*splA*	Serine protease SplA	4.88
RS10530	*chp*	Chemotaxis‐inhibiting protein CHIPS	4.75
RS09610	*splC*	Serine protease SplC	4.40
RS09615	*splB*	Serine protease SplB	4.04
RS09595	*splF*	Serine protease SplF	3.99
RS05720	*hla*	Alpha‐haemolysin	3.81
RS09605	*splD*	Serine protease SplD	3.75
RS10525	*scn*	Complement inhibitor SCIN‐A	3.60
RS15090	–	Phenol‐soluble modulin PSM‐alpha‐3	3.53
RS15730	–	Phenol‐soluble modulin PSM‐alpha‐4	3.50
RS05795	*psm_2*	Beta‐class phenol‐soluble modulin	3.45
RS15735	*–*	Phenol‐soluble modulin PSM‐alpha‐2	3.44
RS00185	*–*	DUF1643 domain‐containing protein	3.29
RS15740	*–*	Phenol‐soluble modulin PSM‐alpha‐1	3.07
RS06445	*glpD*	Glycerol‐3‐phosphate dehydrogenase/oxidase	2.96
RS01705	*gehB*	YSIRK domain‐containing triacylglycerol lipase Lip2/Geh	2.90
RS13080	*hlgB*	Bi‐component gamma‐haemolysin HlgAB/HlgCB subunit B	2.87
RS06715		Hypothetical protein	2.86
RS09600	*splE*	Serine protease SplE	2.83
RS06840	*mucB*	DNA repair protein MucB	2.78
RS00515	*plc*	Phosphatidylinositol‐specific phospholipase C	2.68
RS05790	*psm_1*	Beta‐class phenol‐soluble modulin	2.68
RS02340	*metQ2*	Dipeptide ABC transporter glycylmethionine‐binding lipoprotein	2.63
RS01835	*fepC*	Iron permease FTR1 family protein	2.61
RS06370	*recA*	DNA recombination/repair protein RecA	2.61
RS04005	*uvrA*	Excinuclease ABC subunit UvrA	2.61
RS04395	*ear*	DUF4888 domain‐containing protein	2.57
RS02335	*metP2*	ABC transporter permease	2.53
RS14170	*nrdG*	Anaerobic ribonucleoside‐triphosphate reductase activating protein	2.46
RS04985	*comK1*	Competence protein ComK	2.42
RS13075	*hlgC*	Bi‐component gamma‐haemolysin HlgCB subunit C	2.36
RS10660	*–*	HNH endonuclease	2.34
RS10495		MAP domain‐containing protein	2.30
RS10845	*lukG*	Bi‐component leukocidin LukGH subunit G	2.29
RS01825	*fepA*	EfeM/EfeO family lipoprotein	2.27
RS02330	*metN2*	Methionine ABC transporter ATP‐binding protein	2.15
RS10505	*hlb‐1*	Sphingomyelin phosphodiesterase	2.14
RS04000	*uvrB*	Excinuclease ABC subunit UvrB	2.13
RS14555	*–*	S‐adenosyl‐l‐methionine hydroxide adenosyltransferase family protein	2.13
RS06710	*lexA*	Transcriptional repressor LexA	2.11
RS13060	*sbi*	Immunoglobulin‐binding protein Sbi	2.11
RS10420	*–*	YolD‐like family protein	2.10
RS07540		Panton‐Valentine bi‐component leukocidin subunit F	2.09
RS08875	*infC*	Translation initiation factor IF‐3	2.09
RS04990	*–*	IDEAL domain‐containing protein	2.07
RS03850	*nrdF*	Class 1b ribonucleoside‐diphosphate reductase subunit beta	2.05
RS03840	*nrdI*	Class Ib ribonucleoside‐diphosphate reductase assembly flavoprotein NrdI	2.05
RS11200	*kdpD*	Sensor histidine kinase KdpD	2.04
RS08675	*recJ*	Single‐stranded‐DNA‐specific exonuclease RecJ	2.04
RS06750	*sbcC*	SMC family ATPase	2.04
RS10850	*lukH*	Bi‐component leukocidin LukGH subunit H	2.03

Adjusted *p*‐values for all genes are <0.001.

**Table 2:** Genes significantly downregulated at a fold change <−2 in response to BC.

Locus tag (SAUSA300)	Gene name	Product description	Fold change
RS14135	*betB*	Betaine‐aldehyde dehydrogenase	−6.96
RS01470	*–*	Hypothetical protein	−4.43
RS14005	*–*	TIGR04197 family type VII secretion effector	−3.83
RS09670	*epiA*	Gallidermin/nisin family lantibiotic	−3.78
RS05670	*ecb*	Complement convertase inhibitor Ecb	−3.75
RS02705	*pdxT*	Pyridoxal 5′‐phosphate synthase glutaminase subunit PdxT	−3.66
RS00995	*–*	Alpha‐keto acid decarboxylase family protein	−3.59
RS05755	*argF*	Ornithine carbamoyltransferase	−3.53
RS11395	*ptpB*	Low‐molecular‐weight protein arginine phosphatase	−3.51
RS02700	*pdxS*	Pyridoxal 5′‐phosphate synthase lyase subunit PdxS	−3.35
RS05990	*–*	Hypothetical protein	−3.34
RS14130	*betA*	Oxygen‐dependent choline dehydrogenase	−3.34
RS13660	*ddh*	d‐lactate dehydrogenase	−3.15
RS12650	*–*	DUF805 domain‐containing protein	−3.15
RS02320	*mccA*	Cysteine synthase family protein	−3.14
RS00905	*–*	Hypothetical protein	−3.09
RS11900	*–*	Aldo/keto reductase	−2.95
RS05495	*–*	YlbG family protein	−2.87
RS00665	*–*	MFS transporter	−2.81
RS06680	*katA*	Catalase	−2.80
RS05760	*arcC1*	Carbamate kinase	−2.78
RS11905	*–*	MerR family transcriptional regulator	−2.78
RS05690	*efb*	Fibrinogen‐binding protein	−2.76
RS14030	*–*	Glyoxalase/bleomycin resistance/extradiol dioxygenase family protein	−2.73
RS02920	*sdrD*	MSCRAMM family adhesin SdrD	−2.66
RS01460	*–*	ABC transporter permease	−2.66
RS13140		Type I toxin‐antitoxin system Fst family toxin	−2.62
RS12640	*–*	Sodium ABC transporter permease	−2.61
RS11230	*–*	Hypothetical protein	−2.60
RS00560	*–*	Oleate hydratase	−2.51
RS04765	*–*	DUF2929 domain‐containing protein	−2.50
RS01490	*esxA*	WXG100 family type VII secretion effector EsxA	−2.47
RS13655	*frp*	NAD(P)H‐dependent oxidoreductase	−2.40
RS07865	*–*	DUF1672 domain‐containing protein	−2.40
RS14315	*isaB*	Immunodominant staphylococcal antigen IsaB	−2.36
RS14615	*–*	Arylamine N‐acetyltransferase	−2.34
RS01485	*–*	CHAP domain‐containing protein	−2.31
RS02975	*proP*	Proline/betaine transporter	−2.31
RS01230	*–*	DUF488 domain‐containing protein	−2.25
RS13045	*gpmA*	Phosphoglycerate mutase	−2.24
RS01455	*–*	ABC transporter ATP‐binding protein	−2.22
RS04855	*pepF*	Oligoendopeptidase F	−2.21
RS02030	*nfrA*	NADPH‐dependent oxidoreductase	−2.21
RS08610	*–*	LLM class flavin‐dependent oxidoreductase	−2.17
RS00910	*–*	DUF4242 domain‐containing protein	−2.16
RS01620	*–*	DUF4064 domain‐containing protein	−2.15
RS06940	*–*	Oligoendopeptidase F	−2.15
RS00915	*ssuB*	ABC transporter ATP‐binding protein	−2.15
RS11450	*–*	DUF2529 domain‐containing protein	−2.14
RS02105	*–*	SDR family oxidoreductase	−2.14
RS03315	*mntC*	Metal ABC transporter substrate‐binding protein	−2.14
RS10360	*nadE*	Ammonia‐dependent NAD(+) synthetase	−2.13
RS06665	–	Hypothetical protein	−2.10
RS06380	–	Hypothetical protein	−2.09
RS02970	*–*	HAD family hydrolase	−2.08
RS05620	*trxA*	Thioredoxin	−2.07
RS13480		Hypothetical protein	−2.06
RS04995	*lplA1*	Lipoate–protein ligase	−2.05
RS12320	*fdhD*	Formate dehydrogenase accessory sulfurtransferase FdhD	−2.04
RS09050	*–*	Class I SAM‐dependent methyltransferase	−2.04
RS00990	*–*	Isochorismatase family protein	−2.02
RS02100	*–*	DUF1304 domain‐containing protein	−2.01
RS01240	*hmp*	Nitric oxide dioxygenase	−2.00

Adjusted *p*‐values for all genes are <0.001.

**Fig. 3:**
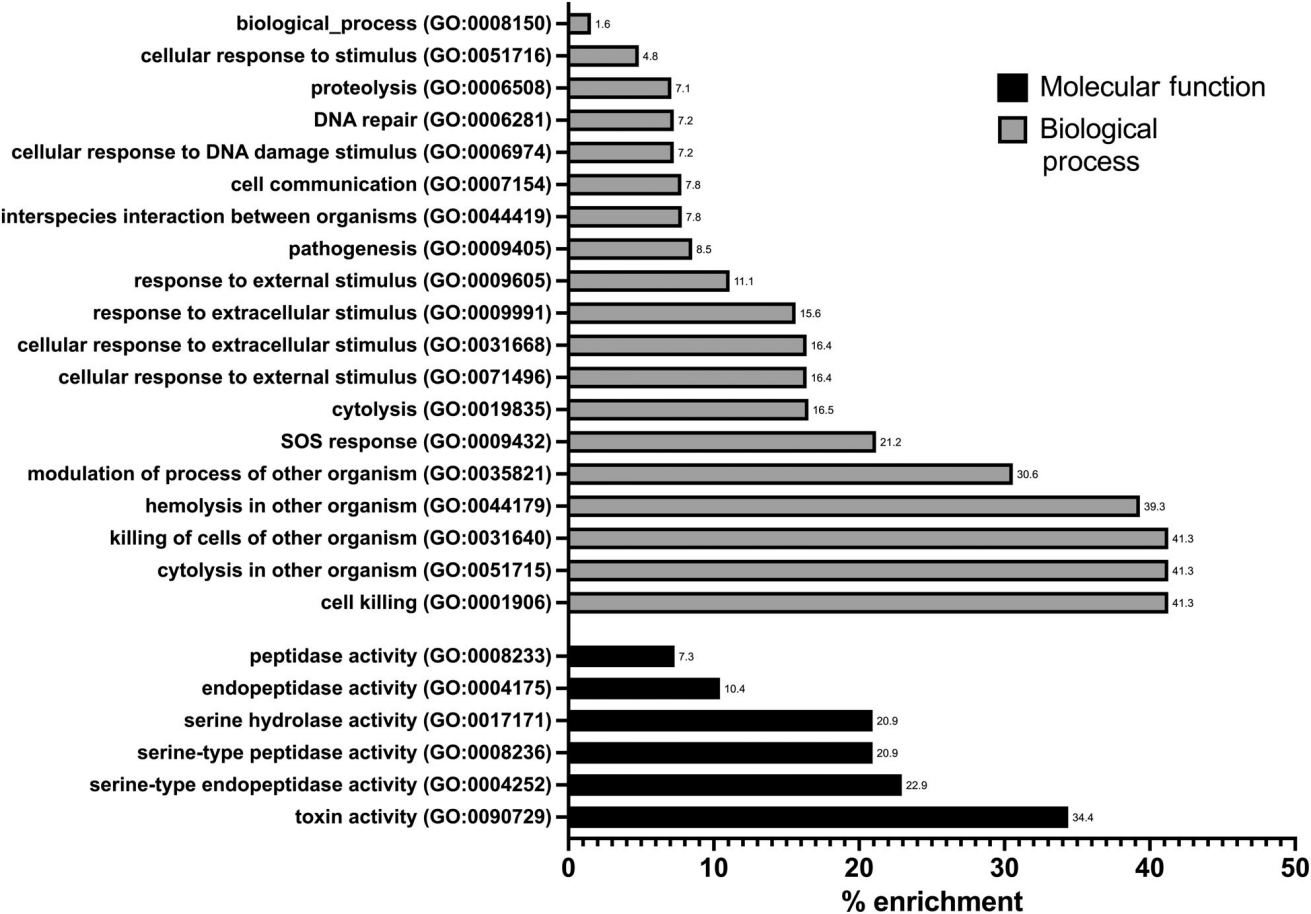
BC exposure results in increased expression of genes involved in toxin production, proteases and DNA replication and repair. Gene ontology (GO) enrichment analysis (A) of 52 genes upregulated in response to BC. The chart shows the over‐representation of each GO term within the dataset as a % enrichment.

Gene ontology (GO) enrichment analysis was used to determine which GO groupings of biological processes (BP), molecular functions (MF) and cellular components (CC) were statistically over‐represented in the presence of BC compared with the absence of BC. The genes upregulated in response to BC showed significant over‐representation of 19 BP and six MF genes (Figure 3). In the BP category the top four terms (cell killing GO:0001906, cytolysis in other organisms GO:0051715, killing of cells of other organisms GO:0031640 and haemolysis in another organism GO:0044179) are all involved in cell killing. This is mirrored in the MF, in that the most overrepresented term is toxin activity (GO:0090729). Other BPs that show over‐representation include those involved in the response to environmental changes and DNA damage and repair. There was no significant over‐representation of any biological processes or molecular functions in the negatively regulated genes and no cellular components in either growth condition.

**Fig. 4:**
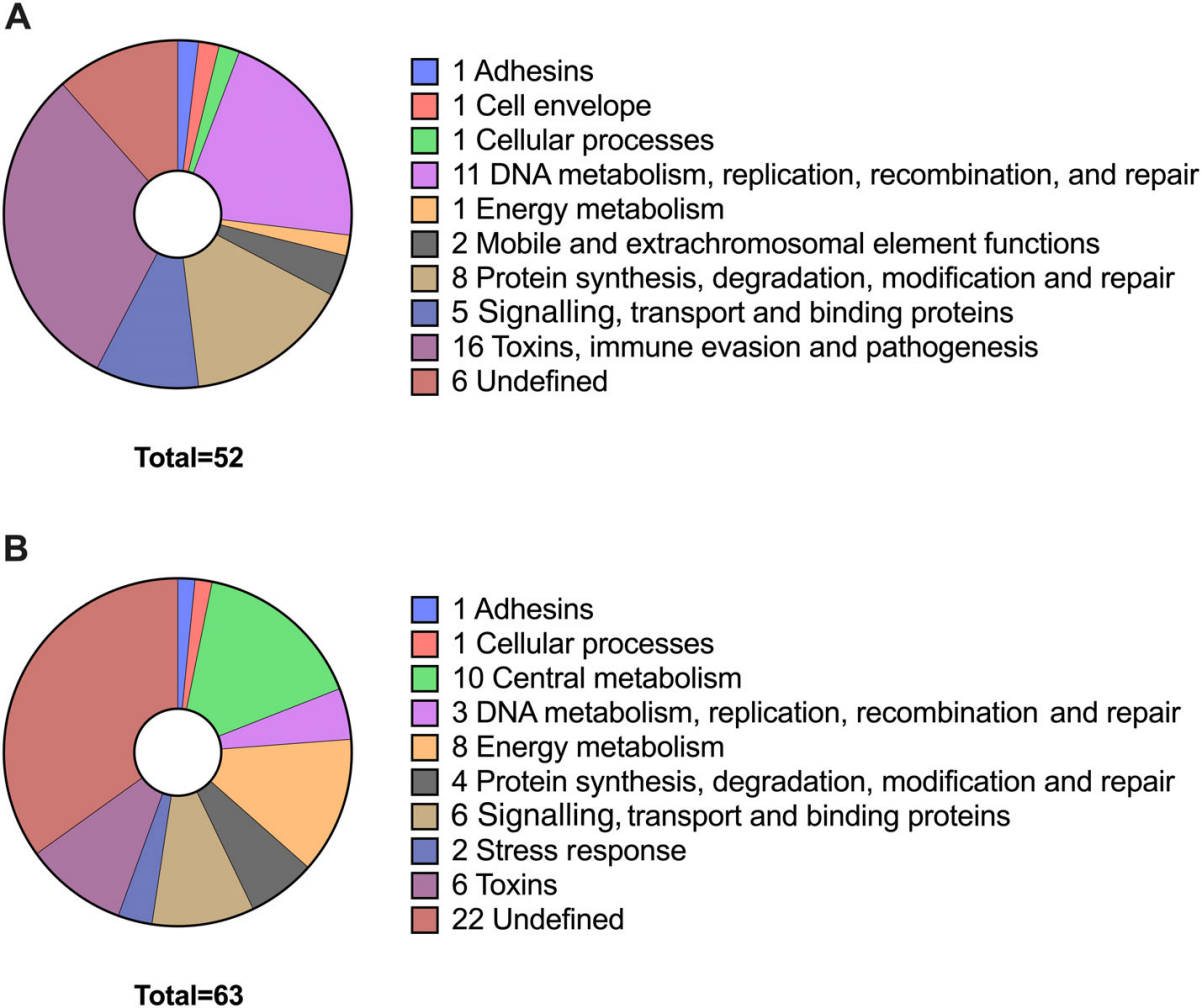
BC exposure results in differential regulation in *S*. *aureus*. Pie chart organizing (A) the 52 upregulated genes and (B) the 63 downregulated genes, by their main TIGRFAM function.

The differentially expressed genes were also grouped based on their main function, using the associated TIGRFAM number which automatically groups proteins, based on sequence homology, into functional families and provides most‐likely functions for hypothetical and unannotated genes (Haft *et al*., 2013; Haft *et al*., 2018) (Tables S2 and S3 and Figure 4). Of the 52 genes upregulated in response to BC, 16 (30.1%) are involved in toxin production, immune evasion, or pathogenesis, 11 genes (21.1%) are involved in DNA metabolism, replication, recombination and repair, and eight genes (15%) play a role in protein synthesis, degradation and repair (Figure 4A). The remaining genes mainly play roles in cellular processes, cell envelope and signalling, transport and binding, and six genes (11%) are currently undefined. Of the 63 genes downregulated in response to BC, the largest represented groups contain 10 genes (15.9%) involved in central metabolism and eight genes (12.7%) involved in energy metabolism, with 22 undefined genes (35%) (Figure 4B).

GO analysis showed that genes associated with pathogenesis are induced by BC. We observed twofold to sixfold induction of the serine protease genes (*splA*, *B*, *C*, *D*, *E*, *F*), and genes for toxins and immune evasion (*hla*, *hlb‐1*, *hlgBC*, *gehB*, *scn*, *chp*, *plc*, *psm alpha‐1*, *2*, *3*, *4*, *psm beta‐1*, *2*, *sbi*, *lukF‐PV*, *lukG*, *H*). It is notable that all these genes are regulated by either the Agr quorum‐sensing system (Bronesky *et al*., 2016; Kavanaugh & Horswill, 2016; Le & Otto, 2015) or the SaeRS two‐component regulatory system (Liu *et al*., 2016; Voyich *et al*., 2009) or both (Table 1).

Several genes involved in DNA repair show a twofold to 2.6‐fold induction in response to BC exposure. These include the genes for the UvrAB nucleotide excision repair endonuclease, the UmuC error‐prone polymerase V and ribonucleotide reductase, HNH endonuclease, Yol‐D family protein and single‐stranded‐DNA‐specific exonuclease RecJ that form an integral part of the SOS response (Podlesek & Žgur Bertok, 2020).

The other genes induced by BC are for glycerol‐3‐phosphate dehydrogenase/oxidase (*glpD*), the FepABC haem utilization system (*fepAC*) (Turlin *et al*., 2013), the dipeptide methionine transporter (*metNPQ2*) (Wade *et al*., 2004) and the potassium transporter and regulator (*kdpA*, *kdpD*) (Xue *et al*., 2011). These genes are regulated by CcpA, the iron repressor protein Fur, the cysteine metabolism regulator CymR and KdpDE two‐component regulator respectively (Fuchs *et al*., 2018; Nagarajan & Elasri, 2007).

### BC represses expression of genes for stress responses and metabolism in *S*. *aureus*

GO analysis did not show any significant over‐representation of downregulated genes, unlike the upregulated genes. Several genes that are repressed in BC are typically induced in response to different stresses, including those involved in oxidative stress (*katA*, *trxA*), osmotic stress (glycine betaine synthesis *betAB*, proline/betaine transporter *proP*), sulfur metabolism (*cysM*, *proP*, *ssuB*) and nitrosative stress (*hmp*, *ldh*).

Exposure to BC also results in repression of genes for some adhesins (*sdrD*, *efb*) and an immune evasion factor (*ecb*). Interestingly, although the stress response (*betAB*, *ldh*, *proP*) and metabolic genes (*argF*, *ptpB*, *pepF*, *nadE*) that are repressed by Agr are also repressed by BC, the majority of the adhesin genes normally repressed by Agr showed no change in expression (e.g. *fnbA*, *fnbB*, *emp*, *spa*; refs), suggesting that the Agr regulon is only partially affected by BC. It is also noteworthy that BC exposure partially repressed the regulons of other global regulators [e.g. CymR (*cysM*), SigB (*proP*), GraRS (*ldh*, *entB*) PdxR (*pdxS*, *pdxT*), RexAB (*frp*, *ldh*), PerR (*katA*, *trxA*) [Fuchs *et al*., 2018; Nagarajan & Elasri, 2007]] showing that BC acts as a signal that induces a newly described pattern of *S*. *aureus* global gene expression.

**Fig. 5:**
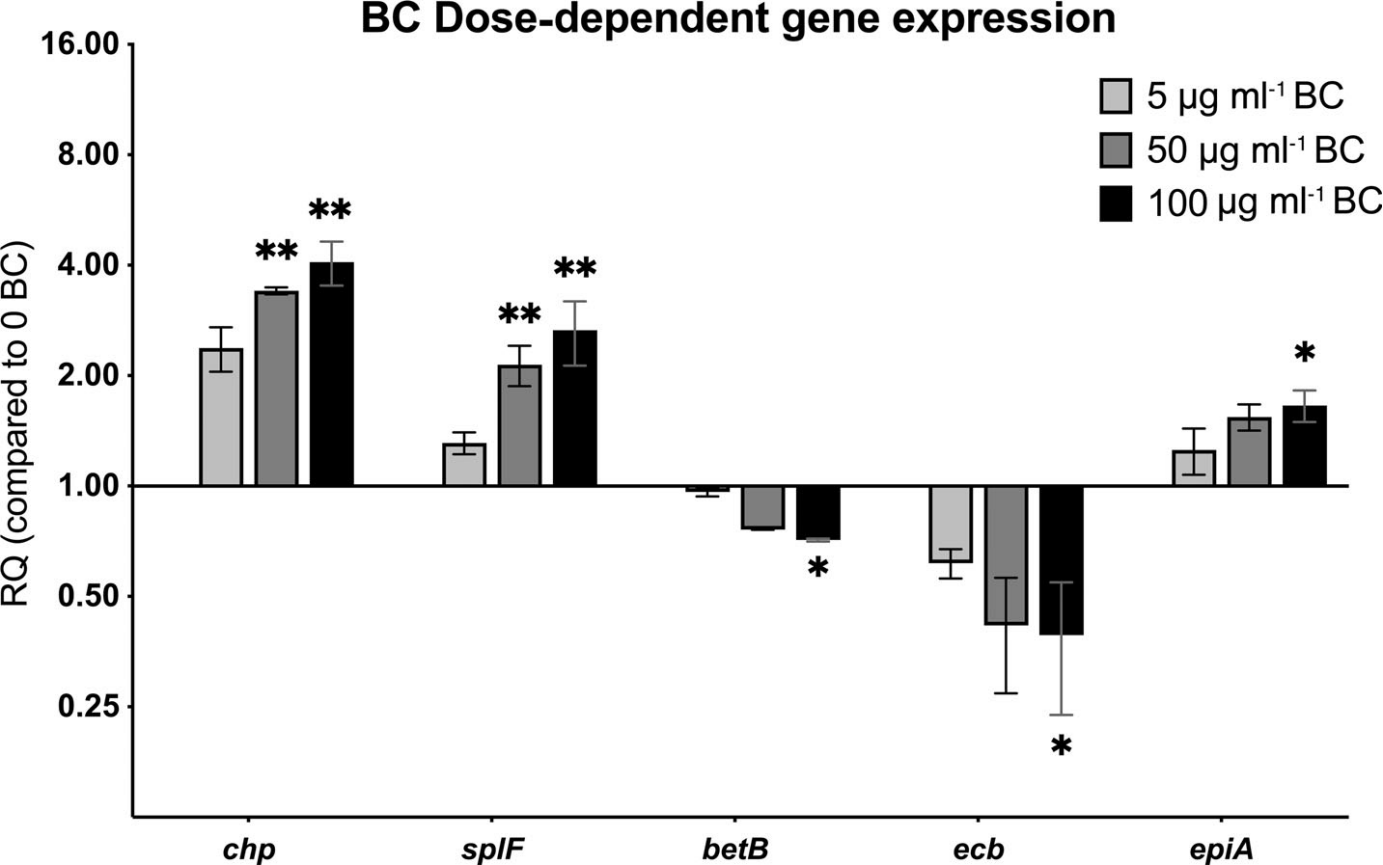
Gene expression changes in response to BC are dose dependent. Relative fold change in *S*. *aureus* USA300 gene expression grown in the presence of 5, 50 and 100 μg ml^−1^ BC. RQ is the fold change in expression relative to –BC. Significance of each concentration compared to 0 BC was determined by Kruskal–Wallis test with Dunn's multiple comparison test (*p < 0.05, **p < 0.01).

To establish whether BC induces gene expression at lower BC concentrations, qRT‐PCR was used to determine *S*. *aureus* gene expression in response to BC at 5, 50 and 100 μg ml^−1^. The expression of the virulence genes *chp*, *splF*, *betB*, *ecb*, and *epiA* all showed a clear concentration‐dependent effect in response to BC (Figure 5) showing that BC can induce *S*. *aureus* gene expression at low concentrations.

### BC‐induced transcriptional changes correspond to increased haemolysis

**Fig. 6:**
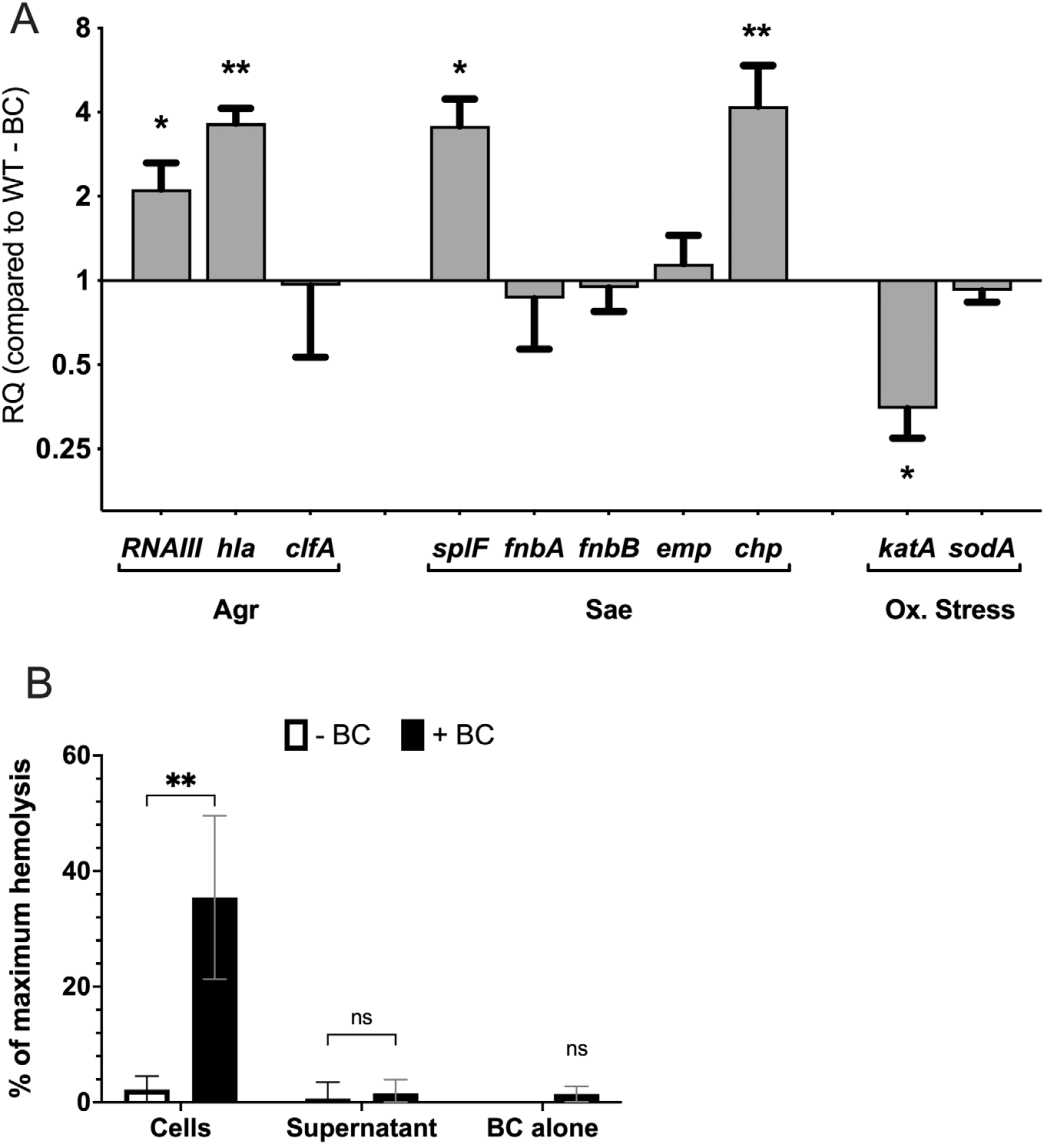
BC induces unexpected patterns of gene expression in key virulence and stress regulons. A. Relative fold change in *S*. *aureus* USA300 gene expression grown in the presence of 100 μg ml^−1^ BC. Effector genes are grouped based on their primary regulator (Agr, Sae or Oxidative stress). RQ is the fold change in expression relative to –BC. Significance of each concentration compared to 0 BC was determined by Kruskal–Wallis test with Dunn's multiple comparison test (*p < 0.05, **p < 0.01). B. Haemolysis activity of *S*. *aureus* USA300 cells and culture supernatant after growth with and without 100 μg ml^−1^ BC. Haemolytic activity was measured as haemoglobin released from prepared rabbit erythrocytes cells after 30 min exposure to *S*. *aureus* cells or supernatant. The data are presented as % of maximum haemolysis and is calculated relative to spontaneous haemolysis (PBS, 0%) and maximum haemolysis of the cells (SDS, 100%). Significance was determined by two‐way ANOVA (*p < 0.05).

The transcriptional analysis showed that BC alters the expression of only subsets of the Agr and Sae regulons. This conclusion from the RNAseq analysis was tested using qRT‐PCR. Of the Agr and Sae responsive genes investigated, there were significant changes in expression of the Agr (*hla*; Figure 6A, *p* < 0.05) and Sae (*chp*; Figure 6A, *p* < 0.01) regulated genes for toxin and immune evasion factors, in agreement with the RNAseq data, but none of the other Agr‐ and Sae‐regulated adhesins (*clfA*, *fnbA*, *fnbB*, *emp*) showed significant change in response to BC (*p* > 0.05), also in agreement with the RNA seq analysis (Figure 6A). The *agrBDCA* and *sae* operons also showed no significant increase in expression in response to BC (data not shown), whereas RNAIII showed a significant twofold increase in expression (Figure 6A, *p* < 0.05). Furthermore, PerR‐regulated oxidative stress genes (Horsburgh *et al*., 2001) also showed differential regulation in response to BC, with decreased transcription of *katA* (Figure 6A, *p* < 0.05) but no change in that of the *sodA* gene. All genes tested showed the same response to BC in *S*. *aureus* grown to mid‐logarithmic or post‐exponential phase (data not shown). Overall, these data confirm the RNAseq analysis and show that BC induces a hitherto unseen pattern of gene expression.

The *hla* gene, which encodes α‐toxin a pore‐forming haemolysin that binds to the membrane of erythrocytes causing haemolysis (Seilie & Bubeck Wardenburg, 2017), showed increased transcription in response to BC (Figure 6A). To establish that induced transcription results in increased toxin activity, the haemolytic activity of either washed bacterial cells or supernatants of *S*. *aureus* LAC grown with and without 100 μg ml^−1^ BC was tested using a rabbit erythrocyte assay. SDS was used as a control to completely lyse the erythrocytes (100% lysis) and haemolytic activity is presented as a percentage of total haemoglobin released. BC and TSB medium were used as negative controls. *Staphylococcus aureus* cells grown in TSB with BC showed a significant increase in haemolytic activity compared to TSB without BC (Figure 6B, *p* < 0.05), whereas BC alone had no effect on haemolysis. Interestingly, we do not see any change in haemolytic activity in the supernatant from the same bacterial cultures (Figure 6B). Thus, BC does indeed cause an increase in toxin activity.

### BC‐induced transcriptional changes require functional Agr and Sae regulators

**Fig. 7:**
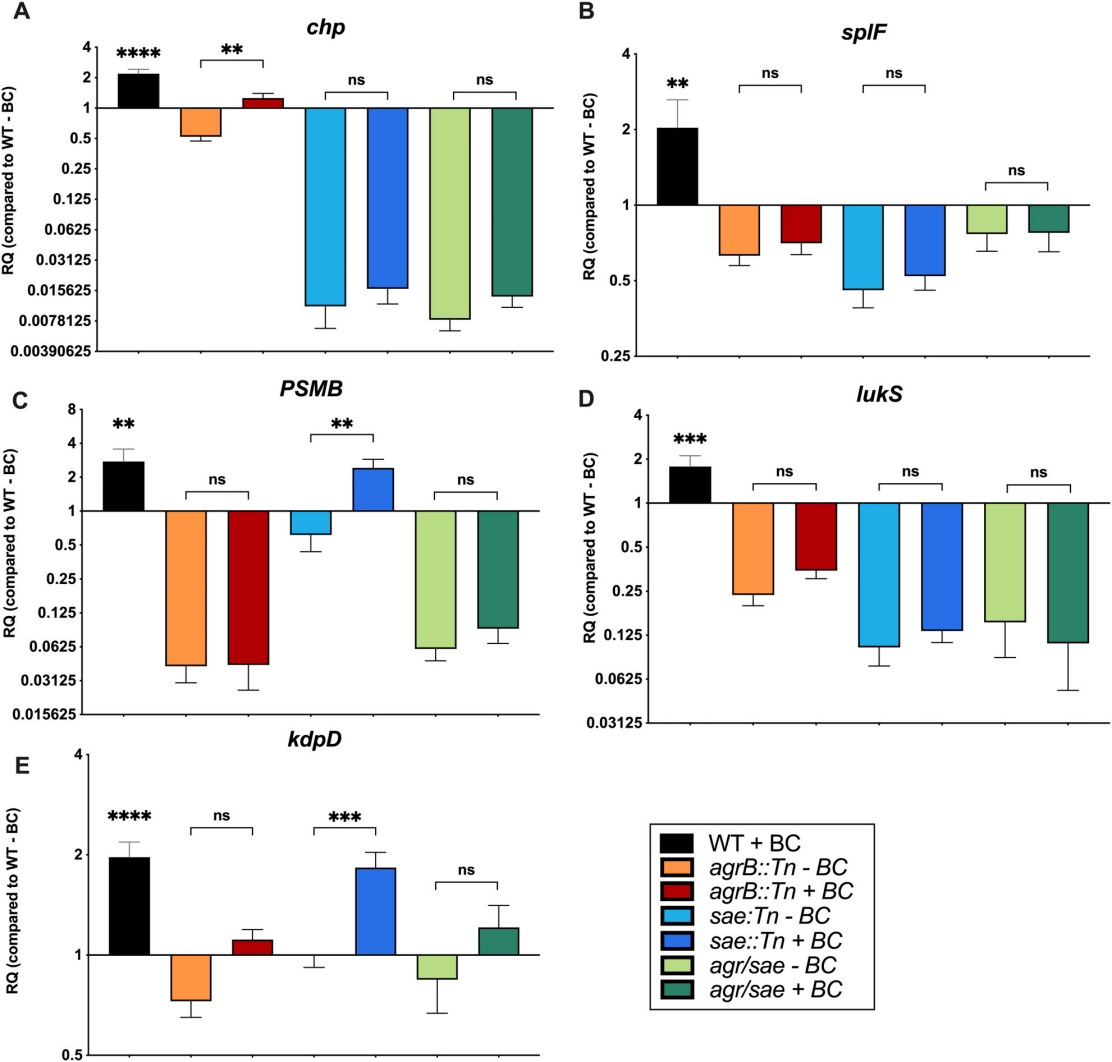
The transcriptional regulators *agr* and *sae* are involved in the BC regulation of some but not all BC‐induced genes. Transcriptional response of *S*. *aureus* genes in response to BC in USA300 LAC WT, *agrB*::Tn, *sae*::Tn and *agr*::tet/∆*saePQRS* mutant strains. RQ is the fold change in expression in each strain relative to the WT – BC. Significance was determined by one‐way ANOVA with Tukey's multiple comparison test (*p < 0.05, **p < 0.01, ***p < 0.001, ****p < 0.0001).

To investigate the role of the Agr and SaeRS regulators in the bacterial response to BC, *S*. *aureus* LAC *agrB* and *saeS* transposon insertion mutants were constructed as described in the methods. With these *S*. *aureus* LAC mutants, the transcription of the Agr‐regulated *psmβ*, the Sae‐regulated *chp* and the dual Agr‐ and Sae‐ regulated *lukS‐PV* and *splF* genes in response to BC was investigated. The expression of *kdpD* was also investigated because *kdpD* is indirectly induced by Agr through repression of the repressor Rot, but it is not regulated by Sae (Xue *et al*., 2011). In the absence of BC, transcriptional analysis confirmed previous studies of Agr and Sae regulation of these genes (Cheung *et al*., 2011; Liu *et al*., 2016). The expression of *lukS* and *splF* show twofold decrease in expression in both the *agr* and *sae* mutants, the *psmβ* gene was not expressed in the *agr* mutant, while *chp* was not expressed in the *sae* mutant, and *kdpD* showed no major change in expression in either the *agr* or *sae* mutants (Figure 7).

Interestingly, in the presence of BC, significant induction of *chp* gene transcription remained in the *agr* mutant (Figure 7A, *p* < 0.01) and *psmβ* and *kdpD* were still significantly induced by BC in the *sae* mutant (Figure 7C, *p* < 0.01; Figure 7E, *p* < 0.001). In contrast, there was no significant BC induction of the *lukS*, *psmβ* or *splF* gene expression in the *agr* mutant or *chp*, *splF* and *lukS* in the *sae* mutant. The involvement of Agr and Sae in response to BC was verified in an *agr/sae* double mutant, which showed decreased expression of the Agr/Sae‐regulated genes (Figure 7). It is noteworthy that these data show that either Agr or Sae are required for BC induction of *chp*, *kdpD* and *psmβ* and that the BC response is facilitated by both genes either together or separately.

### BC induction of *S*. *aureus* epithelial cell invasion is via a sae‐independent mechanism

Our data show that BC increases *S*. *aureus* adhesion and invasion to human epithelial cells. The ability of *S*. *aureus* to adhere to and invade non‐professional phagocytes has been reported to be dependent on Sae induction of the adhesin genes *fnbB*, *fnbA*, *eap* and *atl* (Hirschhausen *et al*., 2010; Liang *et al*., 2006). To investigate the role of Sae and Agr in the BC‐mediated increase in *S*. *aureus* adhesion and invasion, A549 were exposed to wild type *S*. *aureus* LAC, and *agrB* and *sae* mutants pre‐grown in the presence and absence of BC.

**Fig. 8:**
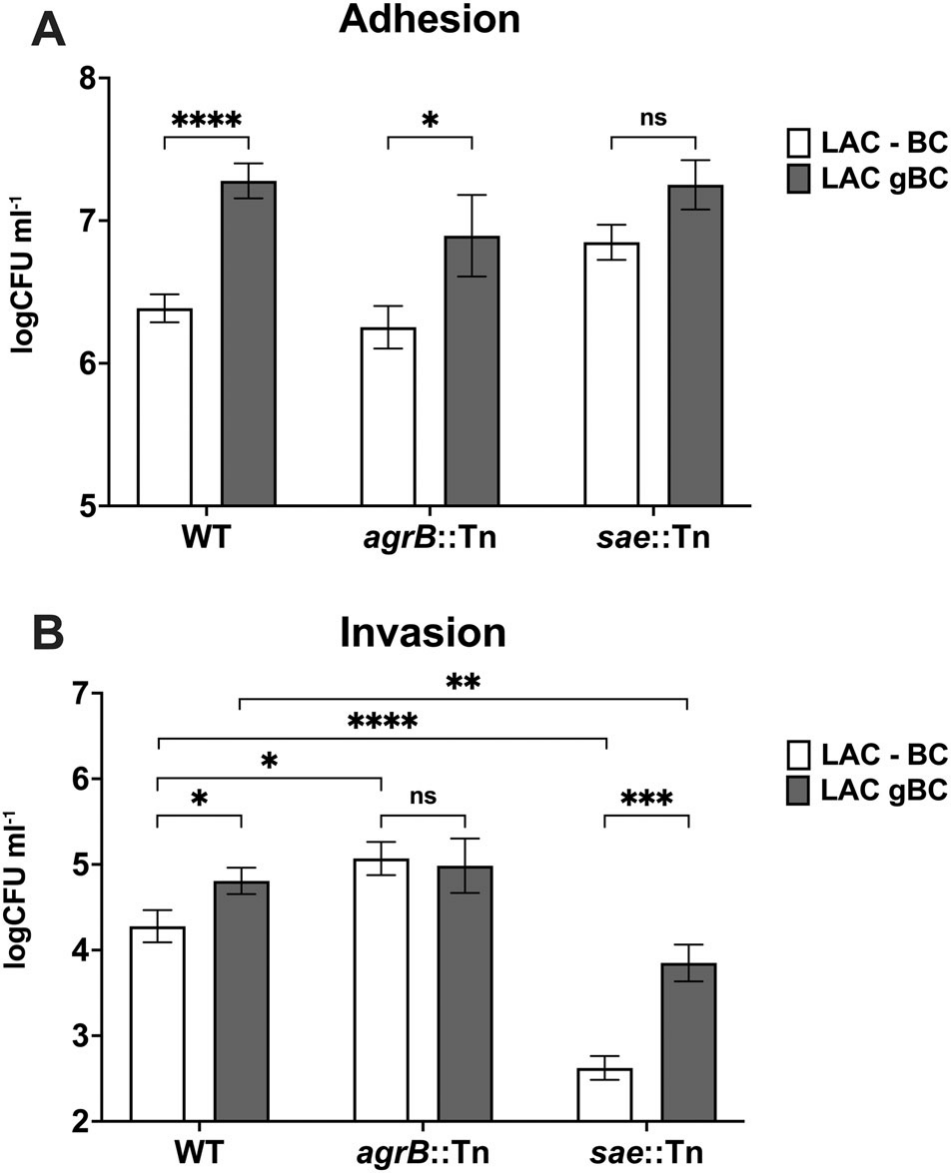
The effect of BC exposure on host cell interaction involves the Sae regulatory system. *Staphylococcus aureus* LAC adhesion (A) and invasion (B) of human lung epithelial A549 cells by *S*. *aureus* LAC WT, *agrB*::Tn and *sae*::Tn mutants in response to BC was measured using a gentamycin protection assay. Cells were infected at a MOI of 100 on 1 × 10^5^ A549 monolayers in 24 well plates. Data are presented as logCFU ml^−1^ of recovered cells, and error bars represent 1 SEM of at least five biological repeats. Significance was determined by two‐way ANOVA with Dunnett's multiple comparison test (*p < 0.05, **p < 0.01, ***p < 0.001, ****p < 0.0001).

As shown in Figure 8, although BC significantly increases *S*. *aureus* LAC adhesion to A549 cells (Figure 8A, *p* < 0.05), neither the *agrB* nor *sae* mutants showed significant changes in adhesion compared to the wild type in the presence or absence of BC (Figure 8A), although the *agr* mutant shows a small decrease in the response compared to LAC and the *sae* mutant. In the absence of BC, the *agr* mutant showed a significant increase in invasion (Figure 8B, *p* < 0.05), whereas the *sae* mutant showed a significant decrease in invasion (Figure 8B, *p* < 0.0001), confirming previous studies of the roles of Sae and Agr in *S*. *aureus* invasion of epithelial cells (Liang *et al*., 2006; Wesson *et al*., 1998). In the presence of BC, there was no significant change in *S*. *aureus* invasion in the *agr* mutant compared to the wild type (Figure 8B). In contrast, there was a significant increase in BC‐induced *S*. *aureus* invasion in the *sae* mutant (Figure 8B, *p* < 0.001) demonstrating that BC mediates staphylococci invasion via Sae and Agr‐independent mechanisms.

## Discussion

BC is a major component of PM in air pollution (Bell *et al*., 2007). Here we show that BC increases *S*. *aureus* colonization of the murine respiratory tract and increases the bacterium's adhesion to human respiratory epithelial cells and its invasion of these cells. Our data show that increased colonization is due to the direct impact of BC on the bacteria and occurs in the absence of any detected BC mediated effects on the host. BC has a widespread effect on *S*. *aureus* global transcription causing increased expression of genes for toxins, proteases, and immune evasion factors critical for dissemination and colonization. Together these data provide evidence for a new causative mechanism of the detrimental effects of air pollution in that air pollutants directly change bacterial gene expression altering their invasive capacity and their ability to colonize and disseminate within the respiratory tract.

There is growing epidemiological evidence that PM exposure increases the risk of infectious diseases that can be caused or exacerbated by *S*. *aureus*; for example, community‐acquired pneumonia, that is increasingly caused by CA‐MRSA (Pivard *et al*., 2021), infective endocarditis (Hsieh *et al*., 2019), cystic fibrosis (Psoter *et al*., 2015; Psoter *et al*., 2017), chronic rhinosinusitis (Schwarzbach *et al*., 2020) and chronic skin diseases (Dijkhoff *et al*., 2020). Exposure to atmospheric PM has great potential to affect the activities of *S*. *aureus* because the bacterium persistently or transiently colonizes the anterior nares and the skin (Pivard *et al*., 2021), where it will be exposed to PM.

In this study we show that simultaneous inoculation of *S*. *aureus* and biologically relevant concentrations of BC causes increased infection of murine lungs and increased nasopharyngeal colonization. These new data agree with our previous observations with *S*. *pneumoniae* exposed to BC (Hussey *et al*., 2017) and confirm a wider phenomenon of the impact of BC on bacterial respiratory tract colonization, that also been recently shown with other types of particulate pollutant (Liu *et al*., 2019; Shears *et al*., 2020; Woo *et al*., 2018; Yadav *et al*., 2020).

It is notable that we showed that pre‐growth of *S*. *aureus* in BC prior to infection induces a significant increase in staphylococcal murine respiratory tract colonization. The increased colonization is maintained for at least 7 days without further administration of BC. To our knowledge, this is the first study to pre‐grow the bacteria with PM prior to inoculation. All previous studies have either pre‐exposed the host to PM or inoculated PM and bacteria together (Liu *et al*., 2019; Shears *et al*., 2020; Woo *et al*., 2018; Yadav *et al*., 2020). These publications hypothesised that increased bacterial colonization was due to PM binding to the bacteria thereby promoting transmission throughout the respiratory tract, or PM providing metabolites to support bacterial growth or PM‐mediated toxicity damaging epithelial integrity (Liu *et al*., 2019; Shears *et al*., 2020; Yadav *et al*., 2020).

In contrast, our data demonstrate a novel explanation of the detrimental effects of air pollution in that BC directly alters bacterial behaviours to increase colonization. Supporting this conclusion, the concentration of BC used in our studies does not have a visible effect on host tissue and does not promote *S*. *aureus* growth (Hussey *et al*., 2017) and murine colonization is promoted even when *S*. *aureus* are pre‐grown with BC and BC has not been directly administered to the mice. BC is a particulate compound that can leach compounds or adsorb solutes from the extracellular milieu, potential chemical and particle effects on bacterial behaviour are currently under investigation in our laboratory. Together these data demonstrate that major effects of particulates on the host are not essential for increased bacterial colonization. Similar conclusions come from the work with A549 cells since there was no detectable impact on A549 cell viability and yet *S*. *aureus* pre‐grown in BC prior to infection show a significant increase in staphylococcal adhesion and invasion of these cells.

RNAseq analysis confirms that exposure to BC alters a collection of *S*. *aureus* genetic responses that have multiple deleterious effects on the host's ability to combat infection and play important roles in colonization and dissemination (Pivard *et al*., 2021). BC increases the transcription of genes for cytotoxins that lyse immune cells (*lukGH*, *hlgBC*, *α* and *β‐psms*) (Collins *et al*., 2020; Tromp & van Strijp, 2020), for factors that inhibit complement (*scin*, *sbi*) (Sultan *et al*., 2018; Pivard *et al*., 2021) and prevent phagocyte recruitment (chemotaxis‐inhibiting protein *chp*), and are important for *S*. *aureus* survival in human blood and neutrophils (phospholipase C, *plc*) (White *et al*., 2014). BC also highly induces expression of the Spl protease genes that play a role in mucin degradation and lung adaptation, with an *spl* mutant showing decreased lung dissemination a rabbit model of pneumonia (Paharik *et al*., 2016).

Interestingly, BC also induces the SOS response regulators (*lexA*, *recA*) and effectors (*uvrAB*, *umuC*, *hnh*, *yolD*, *recJ* and *nrdIFG*). The SOS response is important for the induced expression of genes important for survival and colonization of the host including DNA repair, virulence and immune evasion (Podlesek & Žgur Bertok, 2020). Typically, the SOS response is induced by RecA sensing impairment of bacterial growth and intracellular DNA damage and then initiating the self‐cleavage of the LexA repressor protein (Podlesek & Žgur Bertok, 2020), but effect on growth does not seem to be the trigger here because BC does not inhibit *S*. *aureus* growth and there is no evidence of DNA damage and the transcriptional data do not show other stress responses being activated.

On the contrary, BC represses several genes that are typically induced in response to different stresses, including those involved in oxidative stress (*katA*, *trxA*), osmotic stress (glycine betaine synthesis *betAB*, proline/betaine transporter *proP*), sulfur metabolism (*cysM*, *proP*, *ssuB*) and nitrosative stress (*hmp*, *ldh*) (Fuchs *et al*., 2018; Nagarajan & Elasri, 2007). It must be noted though that the negatively regulated genes do not show such a strong uniform response as the genes induced by BC with there being no significant over‐representation of downregulated genes from any functional group.

The BC induction of the toxin, protease and immune evasion genes (*lukGH*, *hlgBC*, *α and β‐psms*, *hla*, *scin*, *sbi*, *chp*) is likely to occur through the activity of the Agr and Sae two‐component regulators (Cheung *et al*., 2011; Geiger *et al*., 2008) that typically control the expression of these genes. The *S*. *aureus* Agr quorum‐sensing system is important for the switch from a colonizing state to a more aggressive invasive state through induced expression of toxins and the factors required for dissemination (Jenul & Horswill, 2019). Toxin and immune evasion gene expression is also activated by the Sae regulatory system (Geiger *et al*., 2008).

Both Agr and Sae have cell membrane located sensors, the activity of which can be influenced by a range of different environmental conditions (Geiger *et al*., 2008; Kavanaugh & Horswill, 2016), although the exact mechanisms involved have not been fully elucidated. It is possible that BC directly interacts with Agr and Sae by either altering environmental signals such as the Agr quorum‐sensing signal concentrations or activating the membrane‐bound sensors to induce gene expression.

The role of Agr and Sae in BC induction of the toxin and immune evasion genes was confirmed by transcriptional analysis of *sae* and *agr* mutants that showed that both Sae and Agr are associated with BC induction of gene expression. The pattern of response differs between the tested genes with either the Agr or Sae regulator or both being required for BC‐mediated gene regulation. Importantly, BC appears to induce only parts of the Agr and Sae regulons. For example, the expression of adhesin genes that would typically be repressed by Agr (e.g. *spa*) (Cheung *et al*., 2011) and or induced by Sae (e.g. *fnbA*, *fnbB*, *emp*) (Mainiero *et al*., 2010) were not altered in the RNAseq or the qRT‐PCR analysis. Therefore, the data suggest that exposure of *S*. *aureus* to BC prior to or during colonization of the nares would induce a previously unrecognized regulatory response that increases invasive disease, which is distinct from previously described patterns of induction of the Agr and Sae regulons.

BC induction of cytotoxins contrasts with the gene regulatory effects observed with other pollutants, e.g. cigarette smoke extract (CSE). As with BC, CSE increases *S*. *aureus* epithelial cell adhesion and invasion (Kulkarni *et al*., 2012; Lacoma *et al*., 2019; McEachern *et al*., 2015) but in contrast to BC, CSE represses Agr resulting in increased adhesins (Kulkarni *et al*., 2012) and repressed cytotoxin expression (Lacoma *et al*., 2019).

BC caused a significant increase in haemolysis confirming that BC‐induced *hla* transcriptional changes correspond to increased α‐toxin activity. Interestingly, haemolysis only increased when red blood cells were treated with whole cells and not with the growth culture supernatant. This is surprising because α‐toxin is typically secreted and would be expected to be found in the culture supernatant (Seilie & Bubeck Wardenburg, 2017). However, our data suggest that although there is an increase in the level of Hla activity in response to BC, the toxin remains associated with the cell surface of the bacteria rather than being released from the cell. *Staphylococcus aureus* USA300 cell‐associated toxin activity has recently been shown for other toxins with cellular location being dependent on a process that involves the cell membrane lipid, lysyl‐phosphatidylglycerol and lipoteichoic acid (Brignoli *et al*., 2022; Zheng *et al*., 2021). This suggests that BC may influence the cell envelope which could have interesting implications for antibiotic activity.

Furthermore, our data suggest that BC induces a novel mechanism for increased invasion of epithelial cells. Typically, *S*. *aureus* invasion of epithelial cells involves Sae‐dependent mechanisms involving the fibronectin‐binding proteins, lipases and toxin‐induced changes in the cytoskeleton (Josse *et al*., 2017). The only surface proteins showing induced expression in response to BC in the RNAseq data that are not induced by Agr or Sae are the EfeM/EfeO family lipoprotein (*fepA*) and a Map domain protein both of which have no known role in *S*. *aureus* invasion. Therefore, the novel mechanism for *S*. *aureus* invasion induced by BC requires further investigation.

In conclusion, we have provided substantial evidence supporting the novel contention that a single air pollutant, at concentrations non‐harmful to bacteria or the host, can specifically alter bacterial behaviour and would be expected to have adverse health outcomes. This concept has significant implications for mitigating air pollution toxicity and subsequent adverse health effects, because the currently held hypotheses are restricted to the belief that the toxicity of particle pollutants causes adverse effects by damaging the host directly and that control of pollutant levels need only be limited to concentrations below those that are toxic to humans. This study shows that adverse effects can occur at apparently non‐toxic concentrations of pollutant that can alter bacterial behaviour to potentiate infectious disease.

## Supporting information


**Table S1.** Primers used in this study.
**Table S2**. Genes significantly upregulated at a L2FC >1 in response to BC, grouped into TIGRFAM functional groups (Main). * entries do not have an official TIGRFAM entry and have been annotated based on literature review of their functions
**Table S3**. Genes significantly downregulated at a L2FC < ‐1 in response to BC, grouped into TIGRFAM functional groups (Main) * entries do not have an official TIGRFAM entry and have been annotated based on literature review of their functions
**Table S4**. RNA integrity and concentrations of samples sent for RNAseq.
